# The need to accelerate access to new drugs for multidrug-resistant tuberculosis

**DOI:** 10.2471/BLT.14.138925

**Published:** 2015-05-15

**Authors:** Helen S Cox, Jennifer J Furin, Carole D Mitnick, Colleen Daniels, Vivian Cox, Eric Goemaere

**Affiliations:** a Department of Medical Microbiology and the Institute of Infectious Disease and Molecular Medicine, University of Cape Town, Anzio Road, Observatory 7925, South Africa.; b Tuberculosis Research Unit, Case Western Reserve University, Cleveland, United States of America (USA).; c Department of Global Health and Social Medicine, Harvard Medical School and Partners In Health, Boston, USA.; d Treatment Action Group, New York, USA.; e Khayelitsha Programme, Médecins Sans Frontières, Cape Town, South Africa.; f Southern African Medical Unit, Médecins Sans Frontières, Johannesburg, South Africa.

## Abstract

Approximately half a million people are thought to develop multidrug-resistant tuberculosis annually. Barely 20% of these people currently receive recommended treatment and only about 10% are successfully treated. Poor access to treatment is probably driving the current epidemic, via ongoing transmission. Treatment scale-up is hampered by current treatment regimens, which are lengthy, expensive, poorly tolerated and difficult to administer in the settings where most patients reside. Although new drugs provide an opportunity to improve treatment regimens, current and planned clinical trials hold little promise for developing regimens that will facilitate prompt treatment scale-up. In this article we argue that clinical trials, while necessary, should be complemented by timely, large-scale, operational research that will provide programmatic data on the use of new drugs and regimens while simultaneously improving access to life-saving treatment. Perceived risks – such as the rapid development of resistance to new drugs – need to be balanced against the high levels of mortality and transmission that will otherwise persist. Doubling access to treatment and increasing treatment success could save approximately a million lives over the next decade.

## Résumé

On estime à un demi-million le nombre de personnes qui contractent chaque année la tuberculose multirésistante. De nos jours, à peine 20% d'entre elles reçoivent le traitement recommandé, et seulement 10% environ sont traitées avec succès. L'accès limité au traitement est probablement responsable de l'épidémie actuelle qui se propage par une transmission continue. L'amélioration de l'accès au traitement est freinée par les schémas thérapeutiques actuels, lesquels sont très longs, chers, mal tolérés et difficiles à gérer dans les régions où résident la plupart des patients. Bien que de nouveaux médicaments permettent d'améliorer les schémas thérapeutiques, les essais cliniques actuels et prévus ne laissent que peu d'espoir quant au développement de schémas qui favoriseraient l'amélioration rapide de l'accès au traitement. Dans cet article, nous soutenons que les essais cliniques sont certes nécessaires, mais qu'ils doivent être accompagnés d'une recherche opérationnelle de grande ampleur effectuée en temps voulu. Cette recherche permettrait d'obtenir des données programmatiques sur l'utilisation des nouveaux médicaments et schémas tout en améliorant l'accès à un traitement pouvant sauver des vies. Les risques perçus – tels que le développement rapide d'une résistance aux nouveaux médicaments – doivent être mis en balance avec les taux élevés de mortalité et de transmission qui se maintiendraient sans cela. Doubler l'accès au traitement et accroître son efficacité permettrait de sauver environ un million de vies au cours de la décennie à venir.

## Resumen

Se estima que alrededor de 500.000 personas al año desarrollan tuberculosis multirresistente. Apenas el 20% de estas personas recibe un tratamiento recomendado y solo el 10% se somete a un tratamiento eficaz. Probablemente la epidemia actual, a través de la transmisión continua, se deba al escaso acceso al tratamiento. La ampliación del tratamiento se ve obstaculizada por los regímenes terapéuticos actuales, que son prolongados, caros, producen intolerancia y son complicados de administrar en los lugares donde residen los pacientes. A pesar de que los nuevos fármacos son una oportunidad de mejorar los regímenes terapéuticos, los ensayos clínicos actuales y planificados no ofrecen demasiadas esperanzas para desarrollar regímenes que faciliten una ampliación del tratamiento a corto plazo. En este artículo sostenemos que los ensayos clínicos, mientras sea necesario, deberían ir acompañados de una investigación operativa oportuna a gran escala que proporcione información programática sobre el uso de los nuevos fármacos y regímenes, mientras mejora el acceso a un tratamiento que salvará vidas. Los riesgos, como el rápido desarrollo de la resistencia a nuevos fármacos, deberían equilibrarse frente a los altos niveles de mortalidad y transmisión que, de otro modo, continuarán existiendo. Duplicar el acceso al tratamiento y aumentar su éxito podría salvar alrededor de un millón de vidas en la próxima década.

## ملخص

يسود الاعتقاد بأن ما يقرب من نصف مليون شخص يعانون من السل المقاوم للأدوية المتعددة سنويًا. وبالكاد يتلقى 20‏% **  **من هؤلاء الأشخاص حاليًا العلاج الموصى به، وحوالي 10‏% **  **منهم فقط يتم علاجهم بنجاح. ولعل عدم توفر سبل الحصول على العلاج هو ما يؤدي إلى انتشار الوباء الحالي عبر الانتقال المستمر للعدوى. وتتعرقل جهود التوسع في تقديم العلاج بسبب النظم العلاجية الحالية، والتي يشوبها طول المدة والتكلفة العالية وعدم القدرة على تحملها، فضلاً عن صعوبة تقديمها في الأماكن التي يقيم فيها معظم المرضى. على الرغم من أن الأدوية الجديدة توفر فرصة لتحسين نظم العلاج، فإن التجارب السريرية الحالية والمخطط لها لا تقدم سوى القليل من الأمل لإعداد نظم من شأنها تيسير سبل التطوير العاجل لعملية التوسع في تقديم العلاج. ونحن إذ نطرح في هذه المقالة رأينا القائل بأن التجارب السريرية – رغم ضرورتها – إلا أنه ينبغي استكمالها في الوقت المناسب، وعلى نطاق واسع، وإجراء البحث الميداني الذي سيوفر البيانات البرامجية المتعلقة باستخدام أدوية ونظم علاج جديدة مع العمل في نفس الوقت على تطوير سبل الوصول إلى العلاج اللازم لإنقاذ الحياة. ويلزم موازنة الأخطار الملحوظة – مثل التطور السريع لمقاومة الأدوية الجديدة – مع المعدلات المرتفعة للوفيات وانتقال العدوى التي ستستمر بخلاف ذلك. ومن الممكن أن تؤدي مضاعفة فرص إتاحة العلاج وزيادة نسب نجاح العلاج إلى إنقاذ ما يقرب من مليون شخص على مدى السنوات العشرة المقبلة.

## 摘要

每年大约有五十万人被认为患上了多重抗药性结核病。 其中仅有 20% 的人目前接受推荐的治疗方案，而大约只有 10% 的人成功治愈。 治疗不佳便会导致持续性传播，这可能是当前传染病加剧的原因。 治疗范围的扩大受限于当前的治疗方案，该种方案不仅冗长、昂贵、耐受性差，并且很难在多数患者居住的环境中予以实施。 虽然新药让我们有机会改善治疗方案，但是当前和计划的临床试验仍无法保证制定出有助于迅速扩大治疗范围的方案。 在本文中，我们主张临床试验应通过开展及时的、大规模性的操作型研究加以实施，而该种研究须能针对新药和方案的使用提供程式化数据，从而同时改善救生治疗的情况。 感知风险——如新药的抗药性快速产生——需要在高水平的死亡率和传播率之间寻求平衡，否则其将持续存在。 获得加倍治疗并提高治疗成功率，可在未来十年中挽救大约一百万人的生命。

## Резюме

Считается, что ежегодно туберкулезом с множественной лекарственной устойчивостью заболевает около полумиллиона человек. Из этих пациентов только 20% в настоящее время получают рекомендованное лечение, а успешно вылечиваются лишь около 10%. Недостаточный доступ к лечению является вероятной причиной нынешней эпидемии, которая продолжает распространяться. Расширению масштабов лечения препятствуют существующие схемы лечения, которые являются продолжительными, дорогостоящими, плохо переносятся больными и которые трудно проводить в местах проживания большинства пациентов. Несмотря на то что в настоящее время планируются или уже проводятся испытания новых лекарственных препаратов, способных усовершенствовать схемы лечения, эти испытания вряд ли позволят сделать лечение более своевременным в широких масштабах. В данной статье мы приводим доводы в пользу того, что клинические испытания (безусловно, необходимые) должны сопровождаться своевременными, крупномасштабными, операционными исследованиями для получения программных данных о применении новых лекарственных препаратов и лечебных курсов, а также улучшения доступа к жизненно необходимому лечению. Предполагаемые риски, например стремительное развитие устойчивости к новым препаратам, необходимо соотносить с высоким уровнем заболеваемости и смертности, который в противном случае будет сохраняться. Если в следующем десятилетии лечение станет в два раза доступнее и его эффективность будет повышена, потенциально удастся спасти около одного миллиона жизней.

## Introduction

Resistance to antimicrobial drugs threatens incremental gains that have been made in the global fight against tuberculosis. It has been estimated that 480 000 people develop multidrug-resistant (MDR) tuberculosis each year.^1^ In 2013, only 20% of these people received recommended second-line treatment regimens; yet only about 50% of the people that did receive such regimens had successful treatment outcomes.^1^ Therefore, most people with MDR tuberculosis transmit drug-resistant *Mycobacterium tuberculosis* to other members of their communities, since they go undiagnosed or are diagnosed but only treated with ineffective first-line drugs.^2^ There is an urgent need for accessible and improved treatments for MDR tuberculosis – to reduce mortality and avoid scenarios in which resistance becomes the norm rather than the exception. There needs to be a major scale-up of treatment for MDR tuberculosis that matches the scale-ups that have already been achieved in treatments for human immunodeficiency virus and drug-susceptible tuberculosis. The decentralization of diagnosis, care and management is essential. Specialist medical management needs to be largely replaced by nurse-led care, with hospitalization restricted to those patients for whom it is medically indicated. Treatment regimens need to be simplified, shortened and improved, so that treatment success exceeds 50%.^1^

After more than 40 years without any new tuberculosis drugs, there are now several new drugs that are in development or becoming available.^3^ Examples include bedaquiline – which received conditional approval from the United States’ Food and Drug Administration in December 2012 and conditional marketing authorization by the European Medicines Agency in December 2013 – and delamanid – which received conditional authorization by the European Medicines Agency in April 2014. In phase II trials – compared with a placebo and within an optimized background regimen – both of these drugs appeared to be efficacious against MDR tuberculosis.^4^^,^^5^ Phase III trials of these drugs are planned or underway. Some so-called repurposed drugs – e.g. linezolid, which has been primarily used for the treatment of Gram-positive bacterial infections – also appear to be promising in the treatment of MDR tuberculosis.^6^

Despite this apparent progress, access to new or repurposed drugs is associated with substantial challenges for the majority of patients in settings with high burdens of MDR tuberculosis.^7^ In addition to regulatory approval in Europe and the United States of America, specific country-level regulatory approval is often required and can be difficult to obtain. The level of difficulty depends mainly on the sophistication of national intellectual property laws and the regulatory authorities. After approval of a new treatment has been granted, national programmes often grapple with issues of cost and supply and require guidance on appropriate use. In general, such programmes rely on the World Health Organization (WHO) for policy recommendations. Guidance on the appropriate use of new drugs for MDR tuberculosis is currently constrained by limitations in the available evidence, which is often entirely based on the results of phase II and phase III trials in which the efficacies of current regimens with and without a new drug were compared. Much of the available guidance on the optimal use of new or repurposed drugs continues to rely on expert opinion. We need innovative and rigorous observational studies to be run concurrently with – and complementary to – randomized controlled trials. Such studies should be designed to investigate the incorporation of new drugs into more tolerable and practicable regimens and to evaluate the novel regimens under routine conditions.

### Tolerability and practicality

Clinical trials are designed to demonstrate the new drug’s efficacy and safety, for the purposes of obtaining drug approval and registration. They are not designed to identify the best possible treatment regimen to achieve large-scale effectiveness. The approach followed in clinical trials of bedaquiline and delamanid – i.e. adding either drug to an existing, long, toxic, complex regimen – is extremely unlikely to improve treatment access or patient outcomes substantially. Although inclusion of bedaquiline or delamanid has led to improved culture conversion, it has not led to overall treatment success levels that were substantially greater than those reported, in systematic reviews, for the currently recommended treatment regimens for MDR tuberculosis.^4^^,^^5^^,^^8^

Use of the currently recommended regimens is plagued by poor uptake and poor results.^1^ The large pill burden, treatment side-effects and the need for daily injections and routine, long-term hospitalization all lead to high demands on both patients and health systems. As a result, early discontinuation of treatment is consistently above 20% of patients in many settings and up to 52% in some settings.^1^^,^^9^

Compared with any currently recommended regimen, a better-tolerated regimen that can be administered in decentralized settings by the existing tuberculosis programmes should enhance treatment uptake and completion and have a greater impact. An entirely oral regimen – i.e. one that obviates the need for painful, daily injections administered by health workers – would have the combined benefits of improving adherence and treatment completion while reducing severe and debilitating adverse events^10^ and reducing the demands on the health system.

There is a clear need for innovative treatment regimens that incorporate new or repurposed drugs or older drugs in novel combinations that can be used in shorter treatment regimens. The focus of improvements needs to be broadened beyond efficacy and safety to the capacity to scale-up treatment and reach a much larger proportion of the disease burden with a better-tolerated and practical regimen. This approach is relevant for both clinical trials and innovative operational research.

### Clinical trials

The limitations of currently recommended treatment regimens for MDR tuberculosis are largely due to the regimens’ dependence on old drugs, the use of which is generally poorly supported by evidence. It is noteworthy that most companion drugs for such tuberculosis are used off-label (in a manner that, according to the drug’s label, is not formally approved). In contrast to the treatment of drug-susceptible tuberculosis, there have been no clinical trials to guide the design of treatment regimens for MDR tuberculosis. Consequently, drugs have been combined into treatment regimens for MDR tuberculosis based only on expert opinion and observational experience obtained over the past 15 years.^11^ In this context, clinical trials are much-needed.

A phase III trial testing delamanid against placebo is underway, with results expected by 2017.^12^ Both treatment and placebo arms of the trial receive an optimized background treatment regimen. The United States Food and Drug Administration approved bedaquiline under the condition that a phase III trial of the drug be completed by 2022.^13^ Instead of conducting a separate phase III trial, there are plans to incorporate an entirely oral, bedaquiline-containing arm into an existing study (STREAM), which is examining shorter nine-month regimens using existing and repurposed drugs.^14^

In addition to the bedaquiline-containing arms in STREAM, a small number of trials that aim to test combinations of new, repurposed and existing drugs for MDR tuberculosis are being planned. Médecins Sans Frontières, Partners in Health and other groups are planning a set of clinical trials, using adaptive approaches, to design new regimens containing bedaquiline, delamanid and/or the nitroimidazole PA-824 and create shorter, better-tolerated and more effective regimens that have the potential to increase access to treatment in a range of settings.^15^ The Global Alliance for TB Drug Development is implementing a small one-arm study – the so-called Nix-TB trial – to assess a regimen that contains all the new drugs for extensively drug-resistant tuberculosis.^16^ The Global Alliance also plans to enrol a small number of MDR tuberculosis patients in a trial to test a combination of PA-824, moxifloxacin and pyrazinamide.^17^ Unfortunately, the *AIDS Clinical Trials Group’s* plans for a trial of new regimens for MDR tuberculosis^17^ ended in June 2014.

With the exception of the Nix-TB trial, none of the remaining trials that we have mentioned has obtained full approval for implementation or started recruiting patients. Trials to investigate combinations of bedaquiline and delamanid are contingent on the results of a study of drug–drug interaction and safety of the drugs in combination, planned by the United States National Institutes of Health. Although the fast-tracking of this study has been recommended, it remains in preparation and patient recruitment is unlikely to start before late 2015.^18^ Trials combining bedaquiline with PA-824 are also on hold until the safety of the combination can be assessed in the Nix-TB trial. In addition to the delays in planning and implementing clinical trials, clinical trials for tuberculosis are extremely slow. The results of recent tuberculosis trials have only been released 6–9 years after the first patient was allocated to a treatment arm.^19^^–^^21^

Even if the limited number of trials currently in process and planning produce promising results, it is unlikely that we will have data to guide regimen formulation for programmatic use until at least 2020. Given the urgent need for expanded and improved MDR tuberculosis treatment, this timeline appears untenable.

### Policy guidance

WHO released interim policy guidance for the use of bedaquiline in June 2013.^22^ Given the limited data available, WHO recommended that bedaquiline be restricted to tuberculosis patients showing extensive drug resistance and a poor response to their current treatment and to patients affected by adverse events that limit the options for treatment with other drugs. WHO released guidance on the use of delamanid in November 2014.^23^ For delamanid, WHO made similar recommendations as for bedaquiline but also suggested that use of delamanid may not be warranted in situations where an effective regimen can be composed of older drugs. Given the lack of data on the concurrent use of delamanid and bedaquiline, WHO felt unable to make any recommendations on the joint administration of the two drugs. WHO has, however, released general guidance on the introduction of new tuberculosis drugs.^24^ The guidance from WHO is important for national programmes that need to decide how best to utilize the new drugs. However, restricting access to new drugs to specific patient groups in approved, specialist centres – as is currently recommended – fails to meet the need in tuberculosis programmes around the world, where the current availability of treatment for MDR tuberculosis is often very limited .

Bedaquiline is becoming – and delamanid will probably soon become – increasingly available globally. There will soon be growing pressure from clinicians and patients to expand use of both drugs to cover a greater proportion of patients with drug-resistant tuberculosis, both to improve treatment and, potentially, to reduce the side-effects of treatment. The demand for MDR tuberculosis treatment is also likely to increase dramatically as countries expand the use of rapid diagnostic tests.^25^ The fact that patients with drug-resistant tuberculosis in low-burden but high-resource settings are likely to gain access to new drugs will also influence demand. As a result, it is inevitable that both bedaquiline and delamanid will be used in the field to replace existing drugs in a regimen and/or to shorten treatment.

Given the delay in accumulating clinical trial evidence, alternative data to inform the optimal, interim use of these new drugs is required. Such data should increase access to treatment while contributing to the evidence base on use of the new drugs in the settings where treatment is most needed. Evidence could be appropriately incorporated into policy recommendations by WHO and programmatic decisions by countries.

### Operational research

Over the past decade, considerable insights have been made into the development of drug resistance during tuberculosis treatment, along with a greater understanding of the role of pharmacokinetics and drug synergies.^26^^–^^28^ These insights have contributed to choices of drug combinations and regimens that have recently been taken into preclinical studies and clinical trials.^29^ This knowledge can also be used to guide the incorporation of new drugs into existing treatment regimens under operational research conditions, while reflecting specific disease burdens and health system realities. Relatively short (nine- to 10-month) treatment of MDR tuberculosis with existing drugs, based on the regimen already used in Bangladesh,^30^ is currently being assessed in several settings as well as in the STREAM trial. Such short regimens have produced excellent outcomes under operational research conditions in some settings^31^^,^^32^ and may be scaled up before the results of the formal STREAM trial become available. Since the operational research approach to the investigation of existing drugs, as supported by WHO, encompasses ethical review and close monitoring of patients, it could make valuable contributions to the evidence base.^33^

While not using the rigid standards of clinical management and reporting required for clinical trials, observational studies can provide a better quality of care than that available in routine treatment. In order for observational studies to maximize patient safety and provide quality data, they should include: (i) assured access to laboratory and clinical tests, for safety assessment; (ii) strong community-based follow-up, to allow for proactive monitoring of patient outcomes; and, ideally, (iii) a standardized tool for the collection of basic safety and efficacy data (Box 1). While the number of patients included in clinical trials – after satisfying multiple criteria for eligibility – is often in the hundreds, operational studies can aim to treat all diagnosed cases within certain geographical regions and potentially offer treatment to thousands of patients. Instead of the complexities of randomization to different regimens, all patients can be treated with the same regimen. Observational studies can also build local clinical capacity and strengthen health systems – two key prerequisites for treatment scale-up and continued research.

Box 1 **Development of new regimens for the treatment of multidrug-resistant tuberculosis via large-scale operational research projects**

**Key aspects of projects**

- Can include all patients with tuberculosis, including children and those with human immunodeficiency virus infection.
- Aim to increase access to effective care.
- Can employ algorithmic approaches to treatment, based on setting-specific access to diagnostics and drug susceptibility testing.
- Can employ individualized treatment to varying extents, dependent on health system constraints and disease burden.
- Aim to enhance health system capacity to diagnose and treat tuberculosis, including enhancement of routine monitoring of treatment and patient outcomes.
- Can collect data in a thorough and compatible fashion, to inform global policy.

**Proposed requirements**

- Ethical review and oversight to maintain patient safety and clinical standards.
- Treatment delivery following principles of good clinical practice.
- Clear criteria for treatment provision.
- Access to laboratory and clinical tests – i.e. complete blood cell counts, liver function tests and electrocardiograms – for safety assessment.
- Access to trained providers, not necessarily on-site, to interpret and advise on test results.
- Proactive monitoring through community-based care.
- Compatible tools for safety and efficacy monitoring and reporting.
- Communication channels for rapid dissemination of results between operational research sites.

### Risks and benefits

**Fig. 1:**
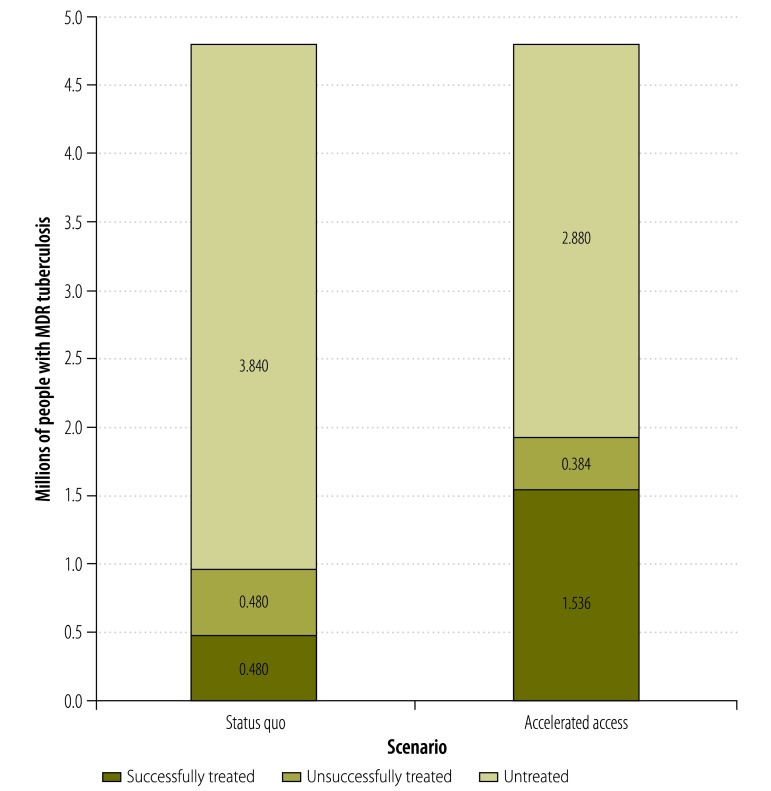
Estimated global number of people with multidrug-resistant tuberculosis, 2015–2024

Treatment as prevention has the dual aims of reducing mortality and interrupting disease transmission. The benefits and risks of accelerated access to new drugs – potentially leading to a greater proportion of patients receiving treatment and a greater treatment success – can be compared with those achieved through a more restricted and gradual introduction. For example, if the incidence of MDR tuberculosis stays the same for another decade, 4.8 million patients will develop MDR tuberculosis.^1^ Doubling access to treatment from the current level of 20% of patients, and increasing treatment success to 80% would result in cure for more than a million additional patients over the next decade (Fig. 1). Given 80% treatment success, tripling access could save 1.8 million lives over the same period. The aim should not be to limit access to new drugs but to facilitate access in a manner that provides the greatest benefit while minimizing risk.

MDR: multidrug resistant.

Notes: The numbers of patients that would occur over the decade that began on 1 January 2015 were estimated using two scenarios. In one – the baseline scenario – the percentage of people receiving the recommended treatment and having a successful treatment outcome were set at the values estimated for 2013: 20% and 10%, respectively.^1^ In the other, so-called accelerated access scenario, these values were increased to 40% and 32%, respectively, to model the possible effects of accelerated access to new drugs. In both scenarios it was assumed that 480 000 persons with rifampicin-resistant tuberculosis would emerge each year. Over the decade investigated there would be 1 056 000 additional persons cured of multidrug-resistant tuberculosis in the accelerated access scenario than in the baseline scenario.

Several researchers have expressed concern that the scaled-up use of new drugs will lead to the rapid development of widespread resistance to the drugs.^34^^,^^35^ This concern motivates efforts to restrict the use of new drugs to small, well-functioning programmes and to use very restrictive criteria for patient eligibility. Current policies emphasize minimizing the risk of widespread resistance. However, this risk needs to be carefully weighed against the deaths that might be expected under different scale-up scenarios. The development of resistance to drugs used to treat tuberculosis and all other infectious diseases is inevitable. Resistance to almost every antimicrobial in clinical use has been detected.^36^ Although resistance to some tuberculosis drugs has emerged rapidly, the mechanisms and drivers of such resistance remain poorly understood.^37^^–^^40^ It also remains unclear if restricting access to a drug slows the acquisition of resistance to that drug.^26^^,^^41^ The acquisition of resistance to the drugs used in the second-line treatment of tuberculosis appears to be driven by regimen composition, such that the risk of such resistance increases substantially as the number of effective drugs in the regimen decreases.^42^ The acquisition of resistance was found to be relatively rare in programmes approved by the Green Light Committee Initiative, probably as the result of strict regimen guidance.^42^ Accelerating access to new drugs – contained in improved, easier-to-administer regimens – may actually reduce the rate at which resistance develops. If treatment outcomes can be improved and the number of patients managed by a nurse or other health-care worker can be increased by the use of simplified, less toxic regimens, the encouraging results seen in programmes approved by the Green Light Committee Initiative ought to be achievable on a much larger scale.

Finally, the safety of a treatment regimen is considered paramount. The maxim of first do no harm can lead to a drug not being used because of rare, but serious, risks to patients. In the case of MDR tuberculosis, no cardiac event has yet been attributed to the use of bedaquiline and delamanid, despite the knowledge that both drugs can prolong the QT interval. However, sudden death, deafness, renal insufficiency, psychosis and irreversible peripheral neuropathy are some of the known adverse effects of the older drugs still used.^43^^–^^46^

## Conclusion

Although the development and approval of new drugs provide hope for the hundreds of thousands of people affected by MDR tuberculosis each year, we still need to know how to use such drugs at scale in the most effective way. If we can rapidly develop a knowledge base on how to use new drugs in combination with existing drugs, we may soon be able to offer effective treatment to all those in need – and not just to those few patients who are able to access specialist care in well-resourced settings.

Scale-up of drug-resistant tuberculosis treatment has been singularly ineffective over the last decade. In the absence of innovative thinking, there is a danger that we will take a conservative approach in which access to new drugs is very restricted and scale-up is slowed. Flexibility and speed – combined with advocacy, political commitment, adequate resources and pragmatism – are now required. 
